# Chiral fermions in asymptotically safe quantum gravity

**DOI:** 10.1140/epjc/s10052-016-4132-7

**Published:** 2016-05-20

**Authors:** J. Meibohm, J. M. Pawlowski

**Affiliations:** 1 Department of Physics, Gothenburg University, 41296 Göteborg, Sweden; 2 Institut für Theoretische Physik, Universität Heidelberg, Philosophenweg 16, 69120 Heidelberg, Germany; 3 ExtreMe Matter Institute EMMI, GSI Helmholtzzentrum für Schwerionenforschung mbH, Planckstr. 1, 64291 Darmstadt, Germany

## Abstract

We study the consistency of dynamical fermionic matter with the asymptotic safety scenario of quantum gravity using the functional renormalisation group. Since this scenario suggests strongly coupled quantum gravity in the UV, one expects gravity-induced fermion self-interactions at energies of the Planck scale. These could lead to chiral symmetry breaking at very high energies and thus to large fermion masses in the IR. The present analysis which is based on the previous works (Christiansen et al., Phys Rev D 92:121501, 2015; Meibohm et al., Phys Rev D 93:084035, 2016), concludes that gravity-induced chiral symmetry breaking at the Planck scale is avoided for a general class of NJL-type models. We find strong evidence that this feature is independent of the number of fermion fields. This finding suggests that the phase diagram for these models is topologically stable under the influence of gravitational interactions.

## Introduction

Finding a well-defined theory for quantum gravity is a major challenge of modern theoretical physics. The asymptotic safety scenario [3] is a promising approach towards a solution to this problem. It relies on the description of quantum gravity in terms of a local, fundamental quantum field theory of the metric. Within this scenario the UV and IR limits of the theory remain well defined but possibly approach strong-coupling regimes where perturbation theory is not applicable.

Non-perturbative functional renormalisation group (FRG) techniques and their application to quantum gravity [4] allow for detailed studies of these strong-coupling regimes. They provide evidence for the existence, and by now also reveal some of the properties of a non-trivial UV fixed point of the renormalisation group flow. The latter controls the UV behaviour of the theory and renders it finite at arbitrarily high energies. For pure gravity such a fixed point was first found in basic Einstein–Hilbert approximations [4–6] and later confirmed in more elaborate truncations [1, 7–25], for reviews see [26–29]. First studies of gravity combined with minimally coupled matter have led to interesting results and developments [2, 30–37]. As a key observation, the non-trivial interplay among the fluctuation dynamics of all involved fields has a crucial impact on the UV behaviour of the theory.

All theories of quantum gravity have to allow for the inclusion of dynamical and potentially (self-)interacting matter [38–42]. In Non-Abelian gauge theories coupled to matter, the gluon-induced fermion correlations are responsible for the generation of fermion masses at low energies. In these theories, the gauge coupling becomes large at low energies and eventually exceeds a critical value. This critical gauge coupling is responsible for induced strong correlations among fermions which lead to chiral symmetry breaking and the generation of fermion masses at low energies. By contrast, gravity becomes strongly interacting in the ultraviolet within the asymptotic safety scenario. This raises the question whether there exists a critical gravitational coupling for which chiral symmetry is broken dynamically at high energies. Crucially, the generation of fermion masses at high energies would result in large masses for fermions in the IR. This is not in agreement with observation, and has to be dealt with in asymptotically safe theories of gravity with matter. The question arises whether there are mechanisms at work that prevent a theory of fermions and gravity from being driven to criticality. One scenario is that the critical coupling is never reached for general initial conditions. In a less restrictive scenario, the system could in principle reach criticality but only for an unphysical set of parameters.

In a previous study [38], the authors find no indications for chiral symmetry breaking in a combined setup of a flat expansion and a background field approach treating the anomalous dimensions of the involved fields as input parameters. In the present work, we reconsider the question of gravity-induced chiral symmetry at energies of the order of the Planck scale on a more general basis in the self-consistent vertex expansion scheme put forward in [1, 2, 7, 8]. In particular, the present setup provides the dynamical couplings (as opposed to the background couplings; see [2]) of the system and allows for the self-consistent calculation of anomalous dimensions. The results are, to a large degree, regularisation scheme independent and also include dynamical anomalous dimensions for all field species. We show that the phase diagram of NJL-type interacting fermion theories is topologically stable under the influence of asymptotically safe quantum gravity. Our study implies that metric gravity and the asymptotic safety scenario stay consistent under the inclusion of an arbitrary number of fermions with a point-like 4-fermion interaction.

## Quantum fluctuations in gravity with fermionic matter

The quantum effective action is the quantum analogue of the classical action and encodes all quantum fluctuations of the theory. In the present case of gravity coupled to fermionic matter we have the effective action $\Gamma [{\bar{g}},\phi ]$ with vertices $\Gamma ^{(n)}$, the amputated *n*-point correlation functions. The effective action depends on a background metric $\bar{ g}$ and the fluctuation field $\phi$, which comprises all fluctuating gravity and matter fields. The full metric field $g_{\mu \nu }$ is split linearly into background and fluctuating fields, $\bar{g}_{\mu \nu }$ and $h_{\mu \nu }$, respectively. For the present theory of gravity and fermions, the complete set of fluctuating fields is given by

1 $$\begin{aligned} \phi =(h,{\bar{c}},c, {\bar{\psi }},\psi ). \end{aligned}$$

**Fig. 1:**
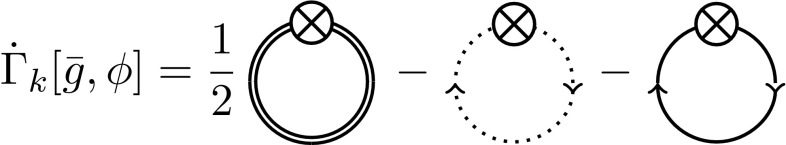
Flow equation for the scale-dependent effective action $\Gamma _k$ in diagrammatic representation. The *double*, *dotted*, and *solid lines* correspond to the graviton, ghost, and fermion propagators, respectively. The *crossed circles* denote the respective regulator insertions

Here, $({\bar{c}},c)$ denote the (anti-)ghosts and $({\bar{ \psi }},\psi )$ are the (anti-)fermion fields. By adding a scale-dependent IR regulator term $R_k[{\bar{g}},\phi ]$ to the classical action we obtain the scale-dependent effective action $\Gamma _k[{\bar{g}},\phi ]$. It gives an effective description of the physics at scale *k* in the spirit of the Wilsonian renormalisation group. The flow of $\Gamma _k[{\bar{g}},\phi ]$ is governed by the Wetterich equation [43], applied to gravity [4]. For the given field content (1) it reads

2 $$\begin{aligned} {\dot{\Gamma }}_k[{\bar{g}},\phi ]&= \frac{1}{2} {\text {Tr}}\left[ \frac{ 1}{\Gamma _k^{(2)}+R_{k}}\dot{R}_{k} \right] _{hh}\nonumber \\&\quad - \text {Tr}\left[ \frac{1}{\Gamma _k^{(2)}+R_{k}}\dot{R}_{k} \right] _{\bar{c} c} {-} \text {Tr}\left[ \frac{1}{\Gamma _k^{(2)}+R_{k}}\dot{R}_{k} \right] _{\bar{\psi }\psi }, \end{aligned}$$

where $\text {Tr}$ denotes the summation over discrete and integration of continuous variables. We abbreviate by a dot derivatives with respect to $t=\log (k/k_0)$, where $k_0$ is some arbitrary reference scale. Figure 1 depicts Eq. (2) in terms of diagrams. From now on, we will drop the regulator insertions in the diagrams as well as the arrows for Grassmann-valued fields for convenience.

Equation (2) cannot be solved for $\Gamma _k[\bar{g},\phi ]$ in full generality. Therefore, the effective action is typically truncated to a finite set of functionals $\mathcal {O}_i$ to wit

3 $$\begin{aligned} \Gamma _k[\bar{g},\phi ]=\sum _{i=0}^N \bar{\alpha }_i(k) \mathcal {O}_i[\bar{g},\phi ]. \end{aligned}$$

With help of this ansatz the flow equation (2) provides a finite dimensional coupled system of flow equations for the *k*-dependent dimensionful couplings $\bar{\alpha }_i(k)$ with mass dimension $d_i$. Dimensionless couplings $\alpha _i$ are introduced by dividing $\bar{\alpha }_i$ with $k^{d_i}$. The flow equations of the dimensionful couplings $\bar{\alpha }_i(k)$ of mass dimension $d_i$ are related to the flow of their dimensionless counterparts $\alpha _i(k)$ by

4 $$\begin{aligned} k^{-d_i} \dot{\bar{\alpha }}_i(k) = d_i \alpha _i + \dot{\alpha }_i. \end{aligned}$$

The truncation applied in this work is constructed along the same lines as in [1, 2, 8]. Thus, $\Gamma _k[\bar{g},\phi ]$ is given in terms of a vertex expansion in the fluctuating fields $\phi$ about the expansion point $\phi =0$ with the structure

5 $$\begin{aligned} \Gamma _k[\bar{g}, \phi ] = \sum _{n=0}^{\infty }\frac{1}{n!}\Gamma ^{(n)}_k[\bar{g},0]\circ \phi ^{n}, \end{aligned}$$

where the circle indicates the integration of $\phi (x_1)\cdots \phi (x_n)$ with $\Gamma ^{(n)}(x_1,\ldots ,x_n)$. As can be seen from (2), the two-point functions plays a pivotal r$\hat{\mathrm{o}}$le in the current approach. We parameterise

6 $$\begin{aligned} \Gamma _k^{(\phi \phi )}(p^2)= Z_\phi (p^2) \mathcal {T}^{(2)}, \end{aligned}$$

where $\mathcal {T}^{(2)}$ carries the tensor structure of the two-point function, see (8), and $Z_\phi$ are the momentum-dependent wave function renormalisations. Similarly, the vertices $\Gamma _k^{(n)}$ are parameterised as

7 $$\begin{aligned} \Gamma ^{(n)}_k[\bar{g},0]&= \left( \prod _{i=1}^{n_h}\sqrt{Z_h(p_{h,i}^2)}\right) \,\left( \prod _{i=1}^{n_c} \sqrt{Z_c(p_{c,i}^2)}\right) \nonumber \\&\quad \times \left( \prod _{i=1}^{n_\psi }\sqrt{Z_\psi (p_{\psi ,i}^2)}\right) \, G_{\vec n}^{\frac{1}{2}(n-2)}\mathcal {T}^{(\vec n)}, \end{aligned}$$

where $\vec n=(n_h,n_c,n_\psi )$ and $n=n_h+n_c+n_\psi$. Here, $n_h$, $n_c$ and $n_\psi$ denote the number of graviton, ghost and fermion legs, respectively. For the two-point functions (7) boils down to (6). A more detailed description of the features of the present truncation can be found in [1, 2, 8]. For $n\ne 2$ the $G_{\vec n}$ are the *k*-dependent couplings of the theory. For example, for the pure gravitational vertices, i.e., $\vec n = (n_h, n_c,0)$ and $n=n_h+n_c$, the $G_{\vec n}$ are momentum-dependent Newton’s constants $G_{\vec n}(\mathbf {p})$. Here, $\mathbf {p}$ is the vector of all field momenta. The vertices that implement the minimal coupling between gravity and fermions are consequently associated with $G_{(n_h,0,2)}(\mathbf {p})$. In the same way as in [1, 2], however, we approximate here all $G_{(n_h,n_c,0)}$ and $G_{(n_h,0,2)}$ as one, momentum-independent coupling, $G_{(n_h,n_c,0)}(\mathbf {p}) = G_{(n_h,0,2)}(\mathbf {p})\equiv G_{(3,0,0)} =: G$. For vertices that involve the 4-fermion interaction we have $G_{(n_h,0,4)}=(G^{n_h}\bar{\lambda }_\psi ^{2})^{1/(n_h+2)}$. If $n_h=0$, the latter expression reduces to $\bar{\lambda }_\psi$, which is the coupling of the pure 4-fermion vertex.

The tensor structures $\mathcal {T}^{(\vec n)}$ are given by the *n*th variation of the classical action *S* to be specified below. The couplings and wave function renormalisations appearing in *S* are replaced by their *k*-dependent counterparts. Bearing this in mind we write the $\mathcal {T}^{(\vec n)}$ as

8 $$\begin{aligned} \mathcal {T}^{(\vec n)}=S^{(\vec n)}\left( \mathbf {p},\Lambda \rightarrow \Lambda _{n}(k),G_N\rightarrow 1, \bar{\lambda }_\psi \rightarrow 1\right) , \end{aligned}$$

where $\Lambda$ is known as the dimensionful cosmological constant and $G_N$ is the classical gravitational constant. We set the classical couplings $G_N$ and $\bar{\lambda }_\psi$ to one since they are replaced by the running couplings $G_{\vec n}$ in the vertex parametrisation (7). The *k*-dependence of all running quantities will be dropped in the following and is understood implicitly. We extract the anomalous dimensions for the fields as well as the flows for all couplings from the flows of the *n*-point vertices. To this end, we substitute our truncation ansatz (5) into the *n*th variation of (2). Evaluating all quantities in a flat metric background $\bar{g}_{\mu \nu }=\delta _{\mu \nu }$ and at vanishing field momenta $\mathbf {p}=0$ we compare coefficients to obtain flow equations for the dimensionful couplings $(G,\Lambda _n,\bar{\lambda }_\psi )$. In a similar manner, we obtain the flows for the wavefunction renormalisations $Z_\phi$, which, however, appear in the flow only in terms of the anomalous dimensions

9 $$\begin{aligned} \eta _\phi (p^2) = -\partial _t \ln (Z_\phi (p^2)). \end{aligned}$$

We achieve the latter simplification by introducing regulators $R^\phi _k(p^2)$, which depend on the momentum-dependent part of the 2-point functions and, consequently, on the wavefunction renormalisation of the respective fields according to

10 $$\begin{aligned} R^\phi _k(p^2) = \Gamma _k^{(\phi \phi )}(p^2)\bigg |_{\Lambda _2=0} r^\phi _k(p^2), \end{aligned}$$

where $r^\phi _k$ denotes the regulator shape function. Details on this general class of regulators can be found in [2].

Consequently, the complete system is governed by the flow of the three couplings given above and a coupled system of equations for the anomalous dimensions $(\eta _h,\eta _c,\eta _\psi )$.

### Classical action

The classical action *S* is given by the sum of the gauge-fixed Einstein–Hilbert action $S_\mathrm{EH}$, which governs the dynamics of classical gravity and the fermionic action $S_\mathrm{ferm}$. The latter contains the fermion kinetic term and describes the classical 4-fermion interactions. Hence, we write *S* as

11 $$\begin{aligned} S=S_\mathrm{EH}+S_\mathrm{ferm}. \end{aligned}$$

The well-known $S_\mathrm{EH}$ reads

12 $$\begin{aligned} S_\mathrm{EH}=\frac{1}{16\pi G_N}\int \left( 2\Lambda -R\right) \sqrt{g}\,\mathrm{d}^4x +S_\mathrm{gf}+S_\mathrm{gh}, \end{aligned}$$

where we denote by $G_N$ the classical Newton coupling. Furthermore, in (12) $S_\mathrm{gh}$ and $S_\mathrm{gf}$ stand for the ghost action and the gauge-fixing action, respectively. The latter are determined by the gauge condition $F_\mu$. In particular, the gauge fixing action reads

13 $$\begin{aligned} S_{gf}=\frac{1}{32\pi \alpha }\int \bar{g}^{\mu \nu }F_\mu F_\nu \,\mathrm{d}^4x. \end{aligned}$$

We apply a de Donder-type linear gauge in the Landau limit of vanishing gauge parameter, $\alpha \rightarrow 0$. More precisely, the gauge-fixing condition is given by

14 $$\begin{aligned} F_\mu = \overline{\nabla }^\nu h_{\mu \nu } - \frac{1+\beta }{4} \overline{\nabla }_\mu {h^\nu }_\nu , \end{aligned}$$

with $\beta =1$ in this work. The Landau limit is particularly convenient since it provides a sharp implementation of the gauge fixing. This ensures that the corresponding gauge-fixing parameter is at a fixed point of the renormalisation group flow [44]. The fermionic action $S_\mathrm{ferm}$ reads

15 $$\begin{aligned} S_\mathrm{ferm} =\int \Bigl (\bar{\psi }^i/\!\!\!\nabla \psi ^i + V(\psi ,\bar{\psi })\Bigr )\sqrt{g}\,\mathrm{d}^4x, \end{aligned}$$

where $V(\psi ,\bar{\psi })$ denotes a fermionic potential with chiral symmetry. Thus, $V(\psi ,\bar{\psi })$ is invariant under the axial rotation

16 $$\begin{aligned} \psi \rightarrow e^{i \alpha \gamma _5}\,\psi \qquad \mathrm{and} \qquad \bar{\psi }\rightarrow \bar{\psi }\, e^{i \alpha \gamma _5 }. \end{aligned}$$

Invariance under (16) also excludes a fermionic mass term. Then the fermion kinetic term is governed by the spin-covariant derivative $/\!\!\!\nabla$ given by

17 $$\begin{aligned} /\!\!\!\nabla =g_{\mu \nu }\gamma (x)^\mu ( \partial ^\nu + \Gamma (x)^{\nu } ). \end{aligned}$$

Here, $\Gamma ^\mu$ denotes the matrix-valued spin connection. The kinetic term is invariant under (16) and, therefore, is chirally symmetric. The operator $/\!\!\!\nabla$ is defined using the spin-base invariant formalism introduced in [45–47] which avoids the use of a vielbein. In particular, it circumvents ambiguities associated with the gauge fixing of the vielbein’s additional Lorentz symmetry [46]. The latter formulation relies on spacetime-dependent spin connection and gamma matrices, which is made explicit in (17). Note that we will drop the spacetime dependence from the notation in the following for convenience.

### NJL-model with one fermion

As a simple, Fierz-complete model for interacting fermions, we consider the NJL-model with one fermion flavour $N_f=1$. Its potential is given by

18 $$\begin{aligned} V(\psi ,\bar{\psi }) =\frac{1}{2}\bar{\lambda }_v \left[ (\bar{\psi }\gamma _\mu \psi )^2\right] -\frac{1}{2}\bar{\lambda }_\sigma \left[ (\bar{\psi }\psi )^2 - (\bar{\psi }\gamma _5\psi )^2\right] , \end{aligned}$$

which is invariant under the axial rotations, (16). While the first term in (18) is trivially invariant due to $\{\gamma _5,\gamma _\mu \}=0$, the scalar and pseudoscalar terms rotate into each other under (16). The first and second terms in (18) represent vector and scalar–pseudoscalar interactions, respectively. We resolve the 4-fermion coupling $G_{0,0,4}$ individually for the two latter interaction channels as $\bar{\lambda }_v$ and $\bar{\lambda }_\sigma$. In the present work we consider momentum-independent $\bar{\lambda }_v$ and $\bar{\lambda }_\sigma$. This ensures that the 4-fermion interactions do not couple back to the minimally coupled sub-system of fermions and gravity which comprises the sub-system of flows $(\partial _t G,\partial _t \Lambda _i,\eta _h,\eta _c,\eta _\psi )$. This crucial feature of the present truncation will be discussed in more detail below.

## Chiral fermions and asymptotic safety

### Chiral symmetry breaking

In this work, we study gravity-induced chiral symmetry at Planck-scale energies. The spontaneous breaking of chiral symmetry occurs if a non-zero expectation value $\langle \bar{\psi }\psi \rangle$ is dynamically generated. This necessitates a divergence of the dimensionless 4-fermion coupling $\lambda$ according to

19 $$\begin{aligned} \frac{1}{\lambda }\rightarrow 0. \end{aligned}$$

**Fig. 2:**
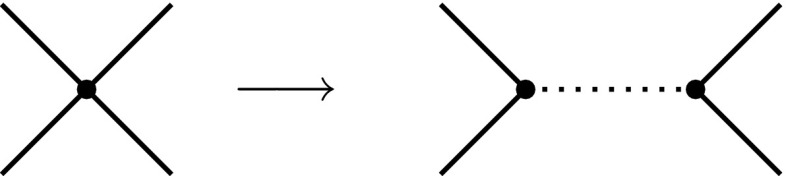
Transition of a point-like 4-fermion coupling to scalar-field-mediated interaction

The origin of this mechanism is most apparent in a partially bosonised description, where the point-like 4-fermion interaction is replaced by the interaction with a scalar field $\varphi$ by means of a Hubbard–Stratonovich transformation. In Fig. 2, this procedure is depicted in terms of diagrams, where the propagation of $\varphi$ is represented by a dashed line. As a result of the transformation, the momentum dependence of the 4-fermion vertex is modelled by the mediation of $\varphi$. The scalar mass $m_\varphi$ is now related to 4-fermion coupling by $\frac{1}{\lambda }\sim {m_\varphi ^2}$. Hence, in this light, Eq. (19) corresponds to a change of sign for the mass term $m^2_\varphi$. This is interpreted as the acquirement of a Mexican-hat shape for the scalar potential which leads to a non-zero vacuum expectation value for $\varphi$ and thus for $\bar{\psi }\psi$.

**Fig. 3:**
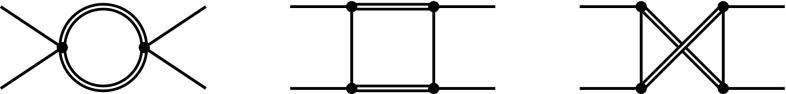
Gravity-induced contributions to the flow of the 4-fermion coupling. The diagrams generate a non-zero 4-fermion self-interaction dynamically, even though the corresponding 4-fermion couplings $\lambda _i$ might be zero at some energy scale

In the following, however, we leave the bosonised formulation aside and study directly the flow of the dimensionless 4-fermion couplings $\lambda _i$ with $i=v,\sigma$ of the potential (18). This allows us to investigate whether chiral symmetry breaking according to (19) is in fact realised in the present model; see e.g. [49–52]. At one-loop level, the 4-fermion interaction is generated by the interaction with gravity via the diagrams given in Fig. 3, and hence is proportional to the Newton coupling squared, $G^2$.

**Fig. 4:**
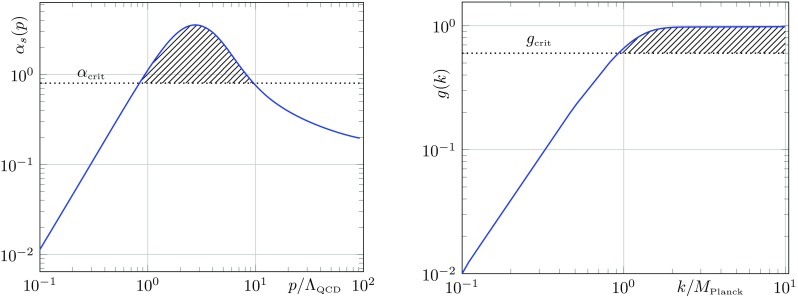
*Left* Running of the strong coupling $\alpha _s$ as a function of the energy scale $p/\Lambda _\mathrm{QCD}$ as calculated in [48]. In order for chiral symmetry breaking to take place, $\alpha _s$ must exceed the critical coupling $\alpha _\mathrm{crit}$ in a sufficiently large interval of $p/\Lambda _\mathrm{QCD}$ (*shaded* region). *Right* Running of the gravitational coupling *g* calculated from the analytical flow equations given in [1]. The *shaded* region represents the interval, where chiral symmetry breaking could take place provided there exists a critical value for *g*(*k*), $g_\mathrm{crit}$, which lies below the threshold of *g*(*k*)

This structure is reminiscent of that leading to chiral symmetry breaking in QCD, where it generates the large low-energy constituent quark masses. In the latter case, the gauge coupling $\alpha _s$ acquires a critical value in the infrared. The left panel in Fig. 4 depicts the running of the strong coupling $\alpha _s$ as a function of the energy scale $p/\Lambda _\mathrm{QCD}$ as calculated in [48].

The analysis in [48] favours a scenario in which $\alpha _s$ becomes critical $\alpha _s(p^2)>\alpha _\mathrm{crit}$ when running towards the IR, but then becomes subcritical again at lower momenta. It is argued that $\alpha _s$ must stay in the critical regime (shaded region) for a momentum scale window that is large enough for chiral symmetry breaking to take place. In the case of gravity, the dimensionless gravitational coupling $g=G k^2$ becomes stronger with increasing RG-scale. Figure 4 shows *g* as a function of $k/M_\mathrm{Planck}$, calculated from the analytical equations in [1] without the inclusion of matter. If a critical coupling $g_\mathrm{crit}$ exists, it is most certainly reached only at $k\approx M_\mathrm{Planck}$. Hence, in contrast to the case in Yang–Mills theories, the existence of a critical coupling for gravity would lead to the generation of fermion masses at energies of the order of the Planck scale. This, however, leads to fermion masses of the order of the Planck scale in the IR, which are not observed in nature.

### Asymptotic safety

It is well known that the NJL-model without gravity interactions exhibits a (Gaussian) IR stable fixed point of the renormalisation group flow. Furthermore, theories of the latter kind have a well-defined UV limit due to the existence of several (non-Gaussian) fixed points with UV stable directions. Thus there exist well-defined limits both for the IR, $k\rightarrow 0$, and the UV, $k\rightarrow \infty$.

On the other hand, there is strong evidence that quantum gravity defined in terms of a quantum field theory of the metric, is asymptotically safe. Therefore, the latter has a well-defined, although strongly coupled UV limit controlled by a UV attractive fixed point. The usual Gaussian fixed point always features one IR attractive direction. Careful studies of the IR behaviour of quantum gravity even suggest the existence of more IR fixed points, apart from the Gaussian fixed point [8]. The purpose of this work is now to analyse the UV and IR behaviour of the— minimally coupled—combined theory of interacting fermions and quantum gravity. The minimal coupling connects the two theories via the kinetic term in the fermionic action $S_\mathrm{ferm}$, which is a function of the fluctuating graviton field *h*.

**Fig. 5:**
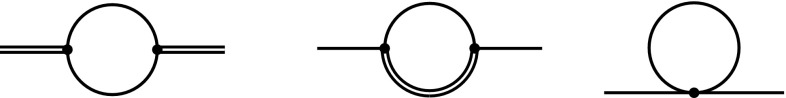
Loop contributions to the flow of the 2-point functions which involve interactions of the fermion field

First, we discuss in which way the two theories interfere due to the minimal coupling. To this end, we neglect the fermion interaction due to $V(\psi ,\bar{\psi })$. In a theory with $V(\psi ,\bar{\psi })=0$ the flows for the graviton and fermion 2-point functions receive mutual loop contributions due to their minimal coupling. The first two diagrams in Fig. 5 depict possible contributions to the latter flows.

The resulting interaction of free fermions with gravity is well known to alter the UV behaviour of the complete theory considerably [2, 12, 35]. However, in all approaches the inclusion of one fermion is no threat to the existence of the UV fixed point. Hence, the combined theory of free fermions minimally coupled to gravity remains well defined.

In the remainder of this work we consider interacting fermions thus $V(\psi ,\bar{\psi })\ne 0$. Note that the 4-fermion interaction along with all 2*n*-fermion interactions are generated dynamically due to the coupling to gravity in the loops. Hence, truncations which disregard the latter interactions cannot capture this part of the flow. The fermion potential (18) discussed in this work includes a Fierz-complete basis of momentum-independent 4-fermion interactions, thereby neglecting the higher 2*n*-fermion vertices.

As mentioned above, non-vanishing 4-fermion interactions $V(\psi ,\bar{\psi })\ne 0$, do not alter the flow of the minimally coupled sub-system of fermions and gravity. This can be understood by considering the possible contributions to the flow of the *n*-point functions that play a rôle here. The 4-fermion interaction enters the flow of the lower order *n*-point functions only via the third diagram in Fig. 5. The latter diagram can only contribute to the fermion anomalous dimension $\eta _\psi$. It does not, however, since the 4-fermion interaction is momentum independent and no external momentum runs in the loop. The resulting decoupling of the flow of the 4-fermion interaction from the rest of the system manifests itself in the fact that the minimally coupled sub-system of fermions and gravity, $(\partial _t G,\partial _t \Lambda _i,\eta _h, \eta _c, \eta _\psi )$, does not depend on the 4-fermion couplings $\bar{\lambda }_\sigma$ and $\bar{\lambda }_v$.

On the other hand, we will see that the flow of the 4-fermion interaction is influenced by the interaction with gravity. Hence, the task is to study which impact the gravity interactions have on the UV and IR behaviour of the 4-fermion theory and, thus, the existence of fixed points of the beta-functions $(\beta _{\lambda _\sigma },\beta _{\lambda _v})=(\partial _t{\lambda _\sigma }, \partial _t{\lambda _v})$ and their stability. The latter are properties of the phase diagram and closely linked to the onset of chiral symmetry breaking during the flow from the UV towards the IR.

## Flow equations

**Fig. 6:**
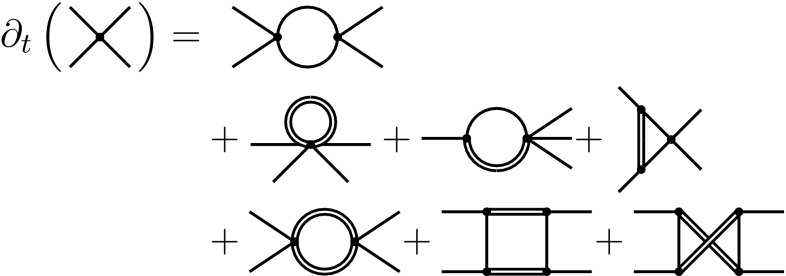
The flow of the 4-fermion coupling given in terms of diagrams. The first, second and third line constitute contributions proportional to $g^0\lambda _i^2$, $g^1\lambda _i^1$ and $g^2\lambda _i^0$, respectively

In terms of diagrams, the flow of the 4-fermion interaction is given in Fig. 6 where we have ordered the diagrams according to the their powers in *G* and $\bar{\lambda }_i$.

The first line of Fig. 6 governs the contributions of the NJL-model without gravity. This part is quadratic in the 4-fermion couplings $\bar{\lambda }_i$ and originates from only one diagram. In the second line we have the contributions linear in *G* and $\bar{\lambda }_i$, several diagrams contribute here. In line three the contributions quadratic in *G* and independent from $\bar{\lambda }_i$ are given. Since the last two diagrams cancel for vanishing fermionic masses, the contribution quadratic in *G* is also governed by only one diagram. Note that another triangle-type diagram with two graviton propagators does in principle contribute to the third line of Fig. 6. However, this vanishes in the here considered Landau limit $\alpha \rightarrow 0$.

From the diagrammatic flow (Fig. 6), we extract the flow equations for the couplings $\bar{\lambda }_\sigma$ and $\bar{\lambda }_v$. In the following, we will express all equations in terms of dimensionless quantities. In particular, the dimensionsless Newton coupling, the dimensionless graviton-mass parameter and the dimensionless 4-fermion coupling are written as $g=G k^2$, $\mu =-2\Lambda _2/k^2$ and $\lambda _i=\bar{\lambda }_i k^2$, respectively. Using the latter, we arrive at the $\beta$-functions for $\lambda _\sigma$ and $\lambda _v$. They read

20 $$\begin{aligned} \beta _{\lambda _\sigma } &= 2\mathfrak {h}\lambda _\sigma ^2+2(1+ \eta _\psi (0)+\mathfrak {f}+4\mathfrak {h}\lambda _v)\lambda _\sigma + 6\mathfrak {h}\lambda _v^2+\mathfrak {g},\nonumber \\ \beta _{\lambda _v} &= \mathfrak {h}\lambda _v^2+2(1+\eta _\psi (0)+\mathfrak {f}+\mathfrak {h}\lambda _\sigma )\lambda _v +\mathfrak {h}\lambda _\sigma ^2-\frac{1}{2}\mathfrak {g}, \end{aligned}$$

which agrees with [51] if we neglect the gravity contributions. Since we evaluate the flow of the 4-fermion vertices at vanishing external momenta, $\eta _\psi (0)$ appears explicitly in the equations. Due to the structure of the 4-fermion interaction we were able to simplify the flow equations considerably, by identifying different terms corresponding to structurally identical diagrams. The functions $\mathfrak {h}$, $\mathfrak {f}$ and $\mathfrak {g}$ correspond to the diagrams depicted in the first, second and third line of Fig. 6, respectively. The quantities $\mathfrak {f}$ and $\mathfrak {g}$ still depend on the gravitational coupling *g* and can be written as $\mathfrak {f}(g)=g \mathfrak {f}(1)$ and $\mathfrak {g}(g)=g^2\mathfrak {g}(1)$. As a result, $\mathfrak {f}$ and $\mathfrak {g}$ vanish in the limit $g\rightarrow 0$. The same is true for the fermion anomalous dimension, $\eta _\psi$. Since $\eta _\psi$, $\mathfrak {f}$ and $\mathfrak {g}$ are the only quantities in our analysis which depend on *g* and $\mu$, these three objects parametrise completely the interaction of the 4-fermion system with the minimally coupled gravity-fermion sub-system.

The functions $\mathfrak {g}$ and $\mathfrak {h}$, correspond to the third and first line in Fig. 6, respectively. Thus, the latter both originate from only one diagram. Since the signs of single diagrams do not change under the change of regulator, $\mathfrak {g}$ and $\mathfrak {h}$ also have fixed signs according to

21 $$\begin{aligned} 0>\mathfrak {g}&= -48\pi g^2\frac{v_3}{(2\pi )^4} \int _0^\infty \frac{\dot{r}_k^{(h)}(q)-\eta _h(q^2) r_k^{(h)}(q)}{(q^2(1 +r_k^{(h)}(q))+\mu )^2}q^3\text {d}q, \nonumber \\ 0>\mathfrak {h}&= -2 \frac{v_3}{(2\pi )^4}\int _0^\infty \frac{\dot{r}_k^{(\psi )}(q)-\eta _\psi (q^2) r_k^{(\psi )}(q)}{(q^2(1 +r_k^{(\psi )}(q)))^2}q^3\text {d}q. \end{aligned}$$

Here, $v_3=2\pi ^2$ is the volume of $\mathbb {S}_3$. By contrast, $\mathfrak {f}$ originates from the three diagrams in the second line of Fig. 6. This means that $\mathfrak {f}$ does in general not have a fixed sign. As we show below, the fixed signs of $\mathfrak {g}$ and $\mathfrak {h}$ allow one to make regulator-independent statements about the fixed points of $(\beta _{\lambda _\sigma },\beta _{\lambda _v})$.

**Fig. 7:**
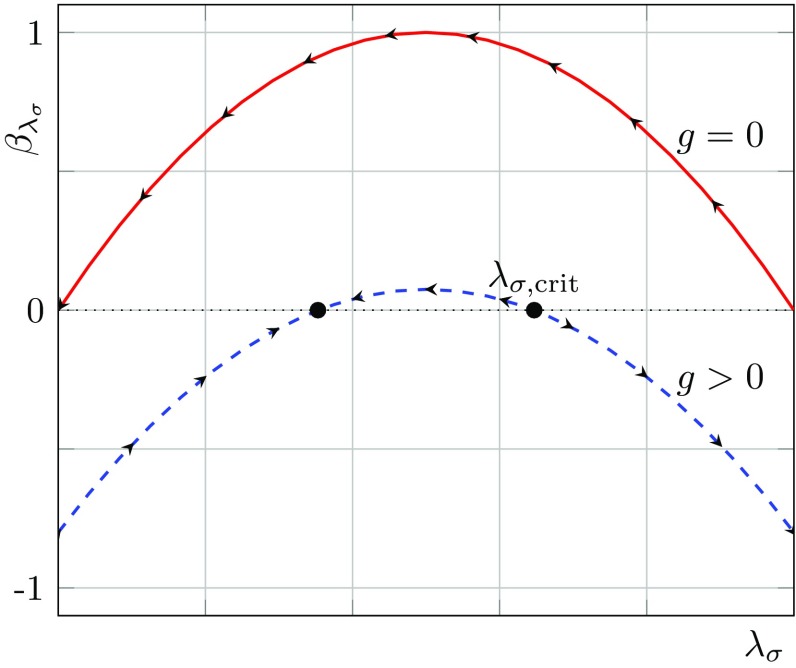
Schematic shape of $\beta _{\lambda _\sigma }$ as a function of $\lambda _\sigma$ for fixed $\lambda _v$, where the *arrows* denote the direction of the flow towards the IR. For $g^*=0$, $\lambda _v=0$ the system exhibits two fixed points. The first one is the IR attractive (Gaussian) fixed point, the second one is IR repulsive. Non-zero $g^*$ or $\lambda _v$ lead to up- or down-shift as well as a deformation of $\beta _{\lambda _\sigma }$

In order to understand the general behaviour of the flows of $\lambda _\sigma$ and $\lambda _v$, we consider first the flow of the scalar–pseudoscalar coupling $\lambda _\sigma$ for fixed $\lambda _v$. For fixed $\lambda _v$ the system of flow equations (20) reduces to only one equation for $\beta _{\lambda _\sigma }$. Since the latter is a quadratic function of $\lambda _\sigma$, it has a generic parabola-shape which is depicted schematically in Fig. 7.

The red solid curve represents $\beta _{\lambda _\sigma }$ for vanishing $\lambda _v=0$ and vanishing dimensionless gravitational coupling $g=G k^2=0$. The dashed blue curve corresponds to the case of non-vanishing $\lambda _v$ and *g*. The $\beta$-function for the reduced system with fixed $\lambda _v$ admits two fixed points (black dots), the arrows represent the corresponding flow in the IR direction. As the arrows suggest, the left fixed point is IR attractive, whereas the right one is IR repulsive. In the present case ($\lambda _v=0$ and $g=0$) the left fixed point is trivial (Gaussian). During the flow towards the IR, the 4-fermion coupling $\lambda _\sigma$ diverges if its value in the UV exceeds the critical value $\lambda _{\sigma ,\mathrm{crit}}$. The latter is given by the value of the repulsive (right) fixed point. In particular, non-zero values of *g*, as given e.g. in Fig. 4, alter $\lambda _{i,\mathrm{crit}}$ in the ultraviolet. Thus, the former result in an up- and down-shift of the solid red curve in Fig. 7, dependent on the sign of the $\lambda$-independent contributions to $\beta _{\lambda _\sigma }$. In (20) the $\lambda$-independent contributions are given by $\mathfrak {g}$ and we have argued above that $\mathfrak {g}$ is negative for all regulators. This suggests a $g^2$-dependent down-shift of $\beta _{\lambda _\sigma }$, represented by the blue dashed curve in Fig. 7. The formerly Gaussian fixed point is driven to non-zero values which results in a non-zero flow for the $\lambda _i$ at $(\lambda _\sigma ,\lambda _v)=(0,0)$. Hence, even if the 4-fermion coupling is zero at some (e.g. cutoff) scale it is always dynamically generated by the flow.

**Fig. 8:**
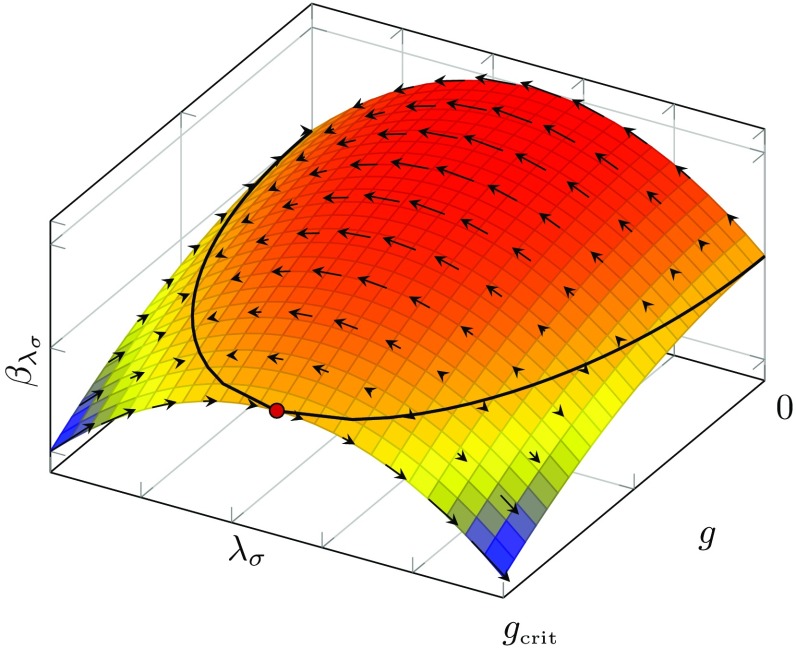
Schematic shape of $\beta _{\lambda _\sigma }$ as a function of $\lambda _\sigma$ and *g* for fixed $\lambda _v$. The *black line* represents the two possible fixed points of the system. For large-enough *g* the two fixed point lines annihilate (*red dot*)

Due to $\mathfrak {g}\ne 0$ the two possible fixed points of the reduced system approach each other and annihilate, if *g* reaches a critical value $g_\mathrm{crit}$. This scenario is in accordance with the assumption that gravity favours strong correlations between fermions and, thus, chiral symmetry breaking. In Fig. 8 the $\beta$-function for $\lambda _\sigma$ is plotted schematically as a function of $\lambda _\sigma$ and *g*.

The black line represents the fixed points as a function of *g*. In the present naïve picture of one 4-fermion coupling the two fixed points annihilate (red dot) if *g* and, hence, $\mathfrak {g}$ are large enough. In this case $\beta _{\lambda _\sigma }$ becomes negative for all values of $\lambda _\sigma$, which is a strong indication for a divergence of $\lambda _\sigma$. In this scenario, the critical value $g_\mathrm{crit}$ is given by the strength of the gravitational coupling *g*, which is necessary in order to result in an annihilation of the fixed points of $\beta _{\lambda _\sigma }$. If *g* stays below $g_\mathrm{crit}$ during the whole RG-flow, the flow of $\lambda _\sigma$ is equipped with well-defined IR and UV limits, $k\rightarrow 0$ and $k\rightarrow \infty$, respectively. In this case the gravitational coupling is not strong enough to drive the 4-fermion interactions to criticality and induce chiral symmetry breaking.

Generally, the requirement that the 4-fermion coupling $\lambda$ exceeds its critical value is a necessary albeit not a sufficient criterion for chiral symmetry breaking. Since the gravitational coupling *g* is a function of the scale parameter *k*, the critical coupling $\lambda _\mathrm{crit}$ changes with the flow as well. This means that it is in principle possible that a quickly varying *g*(*k*) alters $\lambda _\mathrm{crit}$ such that a 4-fermion coupling which had already exceeded its critical value at some scale, say $k_1$, becomes subcritical again at some lower scale $k_2$ with $k_2<k_1$. This behaviour is observed for e.g. Yang–Mills theories as discussed in the previous section (see Fig. 4). The sufficient criterion therefore states that the gravitational coupling *g* stays above its critical value $g_\mathrm{crit}$, which defines $\lambda _\mathrm{crit}$, for a sufficient amount of RG-time $t_\mathrm{crit}=\log (k_1/k_2)$. Within this *t*-interval, $\lambda$ grows rapidly. Thus, if $t_\mathrm{crit}$ is large enough, $\lambda$ grows so large that it exceeds $\lambda _\mathrm{crit}$ at all following RG-times.

For simplicity, however, we will stick for now with the necessary condition of $\lambda >\lambda _\mathrm{crit}$ given above, not taking into account the time spent in the critical $\lambda$-regime. Clearly, if the necessary condition for the onset chiral symmetry breaking is not fulfilled, there is no need to consider sufficient conditions.

**Fig. 9:**
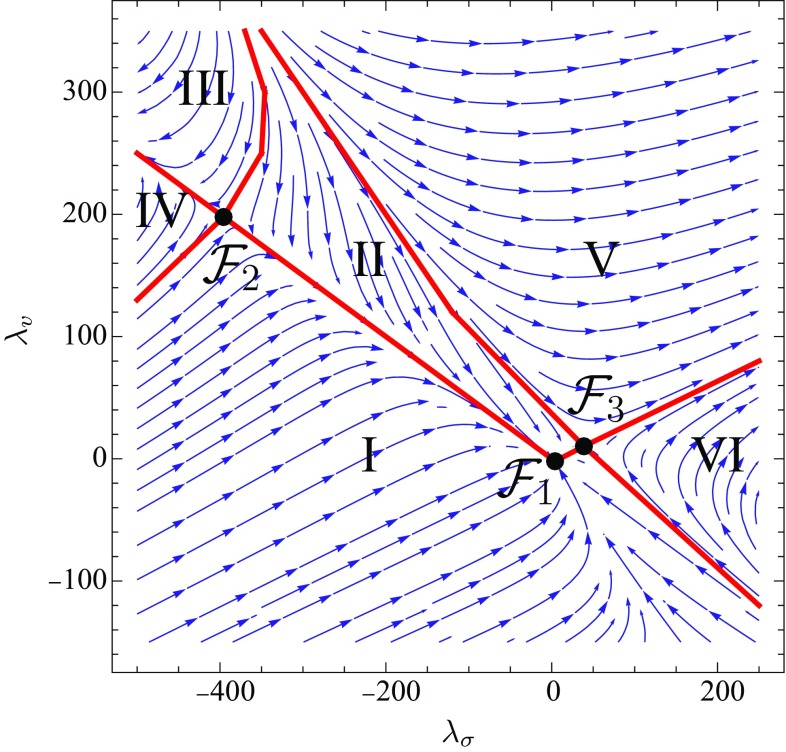
Phase diagram for the couplings $(\lambda _\sigma ,\lambda _v)$ for the values $(g,\lambda _3,\mu )=(1,0.25,-0.5)$ and vanishing anomalous dimensions $\eta _\phi =0$, using a Litim-type shape function, [53]. The *arrows* point towards the IR. The *black dots* denote the fixed points, $\mathcal {F}_1$, $\mathcal {F}_2$ and $\mathcal {F}_3$ of the flow. All trajectories starting in the regions *I* and *II* flow towards the fully IR attractive (almost) Gaussian fixed point $\mathcal {F}_1$

We now investigate the correspondence between the annihilation of fixed points and well-defined UV and IR limits of the theory for the full system $(\beta _{\lambda _\sigma },\beta _{\lambda _v})$. In Fig. 9 the phase diagram of the latter is shown for some hypothetical UV values of the theory with $(g,\lambda _3,\mu )\ne (0,0,0)$.

The arrows represent the flow towards the IR. The values for *g*, $\lambda _3$ and $\mu$ are set to $(g,\lambda _3,\mu )=(1,0.25,-0.5)$ to demonstrate the generic behaviour of the flow of $(\lambda _\sigma ,\lambda _v)$ for non-trivial values of the gravitational couplings. The fermion anomalous dimensions are set to zero for the plot, $\eta _\phi =0$. However, the general case $\eta _\phi \ne 0$ is considered in the rest of the work. For the computation of the phase diagram, we use a Litim-type shape function $\sqrt{x}\,r^{\psi }_k(x)=(1-\sqrt{x})\theta (1-x)$. The NJL-model admits three fixed points $\mathcal {F}_1$, $\mathcal {F}_2$ and $\mathcal {F}_3$. For $g=0$, $\mathcal {F}_1$ is the Gaussian fixed point. However, in direct analogy to the picture given for the reduced system in Fig. 7, the gravitational interaction shifts $\mathcal {F}_1$ slightly to non-zero values. The fixed point $\mathcal {F}_1$ is the only one which is fully IR attractive. The other ones, namely, $\mathcal {F}_2$ and $\mathcal {F}_3$ both exhibit one attractive and one repulsive direction. The fixed-point structure of the NJL-model divides the phase diagram in Fig. 9 into six different regimes that are separated by separatrices marked in red. Trajectories starting in the regimes $\text {I}$ and $\text {II}$ end up in $\mathcal {F}_1$. The fact that the model exhibits only three instead of four fixed points is a curiosity of the NJL-model in $d=4$ spacetime dimensions [51]. One can think of the fourth fixed point as being located at infinity at the point where the separatrices that separate the regimes III and V from II, respectively, intersect.

In contrast to the simplified model of one flow equation discussed above, for the complete NJL-model the non-vanishing of fixed points is *not* a sufficient criterion for the existence of well-defined UV and IR limits any more. Due to the increased dimensionality of the phase space $(\lambda _\sigma ,\lambda _v)$ the fixed points can lose their UV and IR attractivities, respectively *without* simultaneous fixed-point annihilation. Hence, it is feasible that the fixed points persist but their UV and IR attractiveness is flipped by the gravitational interaction. If, say, the formerly Gaussian fixed point $\mathcal {F}_1$ loses its IR attractivity in at least one direction, this changes the topology of the resulting phase diagram, possibly spoiling the well-defined IR limit of the theory.

Hence, the analysis of the phase diagram boils down to two steps. First, we analyse whether interacting gravity leads to an annihilation of fixed points. Leaving the gravity-dependent quantities $(\mathfrak {f},\mathfrak {g},\eta _\psi )$ as external input parameters we will show that the annihilation of fixed points is impossible for any combination of $(\mathfrak {f},\mathfrak {g},\eta _\psi )$, once the constraints (21) are imposed.

In the subsequent second step, we analyse, how the fixed points’ eigenvalues, i.e., their attractivity, change under the influence of gravitational interactions. In particular, we are interested if the signs of the latter can change for certain combinations of $(\mathfrak {f},\mathfrak {g},\eta _\psi )$. We will find that the signs of the eigenvalues crucially depend on the sign of the sum $\mathfrak {F}=1+\eta _\psi (0)+\mathfrak {f}$. This sign, however, is not fixed by (21). Therefore, we perform a detailed analysis of the flow and investigate in which regime of the relevant phase space, $\mathfrak {F}$ does change sign. For this analysis we reduce the system by identifying $\lambda _3=-\frac{1}{2} \mu$. Imposing certain regulator constraints on the anomalous dimensions $\eta _\psi$ and $\eta _h$ we will be able to show that a change of sign of $\mathfrak {F}$ lies either outside the physical regime of the reduced phase space $(g,\mu )$ or outside the regime where the truncation is reliable.

From this two-step analysis we will draw the conclusion that gravity-induced chiral symmetry breaking is absent in interacting 4-fermion models of the NJL-type.

### Fixed-point annihilation

In order to study the fixed points for the NJL-model in full generality, we solve the fixed-point equation, Eq. (20) = 0, for $(\lambda _\sigma ,\lambda _v)$. Remarkably, the fixed points can be given in a simple, closed form which reads

22 $$\begin{aligned} \mathcal {F}_{1,2}&=\left( \frac{2\mathfrak {F}\mp \sqrt{2 \mathfrak {g}\mathfrak {h}+4 \mathfrak {F}^2}}{\mathfrak {h}}, \frac{-2\mathfrak {F}\pm \sqrt{2 \mathfrak {g}\mathfrak {h}+ 4 \mathfrak {F}^2}}{2 \mathfrak {h}}\right) , \nonumber \\ \mathcal {F}_3&=\left( -\frac{2 \mathfrak {g}\mathfrak {h}+3\mathfrak {F}^2}{8 \mathfrak {F}\mathfrak {h}},-\frac{-2 \mathfrak {g}\mathfrak {h}+\mathfrak {F}^2}{8 \mathfrak {F}\mathfrak {h}}\right) , \end{aligned}$$

where, again, $\mathfrak {F}=(1+\eta _\psi (0)+\mathfrak {f})$. Note, that the fixed points of the NJL-model without gravity are recovered by setting $g\rightarrow 0$ and, correspondingly, $\mathfrak {F}\rightarrow 1$ and $\mathfrak {g}\rightarrow 0$. In this case, $\mathcal {F}_1\rightarrow (0,0)$, which means that it is the Gaussian fixed point in this limit. Clearly, the fixed points $\mathcal {F}_1$ and $\mathcal {F}_2$ annihilate, if the argument in the square roots of the first line in (22) becomes negative. The third fixed point $\mathcal {F}_3$ is present for any combination $(\mathfrak {f},\mathfrak {g},\eta _\psi )$. Thereby, the condition for the annihilation of fixed points translates into the simple inequality

23 $$\begin{aligned} \underbrace{\mathfrak {g}\mathfrak {h}}_{>0}+\underbrace{2 (1+\eta _\psi (0)+\mathfrak {f})^2}_{\ge 0}\overset{!}{<}0. \end{aligned}$$

Due to the fixed signs of $\mathfrak {g}$ and $\mathfrak {h}$ given by the Eq. (21), the product $\mathfrak {gh}$ is always positive. This suggests that neither of the two terms in (23) becomes negative. Therefore, the inequality (23) is not fulfilled for any choice of regulator.

In particular, the fermion anomalous dimension at vanishing momentum $\eta _\psi (0)$ as well as $\mathfrak {f}$ do not play any rôle for the sign of left-hand side of (23) since they enter quadratically. We have shown that the annihilation of fixed points due to gravitational interactions in our model is impossible. The only possible way to destroy the well-defined limits now, is via the change of stability of the fixed points.

### Stability of fixed points

We now turn to our second criterion, the stability of the fixed points in the NJL-model. It is conceivable that, if the gravitational interaction is strong enough, it might change the attractivity of its fixed points. In the vicinity of fixed points, the flow can be linearised. Thus, its behaviour is described by critical exponents $\theta _n$, which are the eigenvalues of the matrix $\left( \frac{\partial \beta _{\lambda _i}}{\partial \lambda _j}\right)$ to wit

24 $$\begin{aligned} \theta _n=\text{ Spec }\left[ \left( \frac{\partial \beta _{\lambda _i}}{\partial \lambda _j}\right) \bigg |_{\mathcal {F}_n}\right] . \end{aligned}$$

Hence, from the flow equations (20), together with the fixed-point values (22) we find the critical exponents of the NJL-model with gravity. They take the simple form

25 $$\begin{aligned} \theta _{1}&=\left\{ 6 \mathfrak {F}-4 \sqrt{\mathfrak {F}^2+ \frac{\mathfrak {h}\mathfrak {g}}{2}},2\sqrt{ \mathfrak {F}^2+\frac{\mathfrak {h}\mathfrak {g}}{2}}\right\} , \nonumber \\ \theta _2&=\left\{ 6 \mathfrak {F}+4 \sqrt{ \mathfrak {F}^2+\frac{\mathfrak {h}\mathfrak {g}}{2}}, -2 \sqrt{\mathfrak {F}^2+\frac{\mathfrak {h}\mathfrak {g}}{2}}\right\} , \nonumber \\ \theta _3&=\left\{ \frac{\mathfrak {F}^2+2 \mathfrak {h}\mathfrak {g}\pm \sqrt{81 \mathfrak {F}^4+4 \mathfrak {h}\mathfrak {g}(\mathfrak {h} \mathfrak {g}-7\mathfrak {F}^2)}}{4 \mathfrak {F}}\right\} . \end{aligned}$$

Considering again the limit of vanishing gravitational coupling $g\rightarrow 0$, thus $\mathfrak {F}\rightarrow 1$ and $\mathfrak {g}\rightarrow 0$, we get $\theta _1=\{2,2\}$, $\theta _2=\{10,-2\}$ and $\theta _3=\{5/2,-2\}$ which we know from the NJL-model without gravity. Note that these quantities are regulator independent in the limit $g\rightarrow 0$, since the fermion anomalous dimension does not depend on the 4-fermion couplings. Hence, this fact is a consequence of the momentum-independent 4-fermion interactions considered here. For $g\ne 0$ however, the regulator-dependent quantities $\mathfrak {F}$, $\mathfrak {h}$ and $\mathfrak {g}$ enter the Eq. (25). From these equations we can see that naïvely, the $\theta _i$ could flip sign, if $\mathfrak {F}$ becomes negative. Since $\mathfrak {F}=1+\eta _\psi (0)+\mathfrak {f}$ and neither $\eta _\psi (p^2)$ nor $\mathfrak {f}$ have a definite sign, the case $\mathfrak {F}<0$ is not excluded generally. However, since $(\eta _\psi ,\mathfrak {f})\rightarrow (0,0)$ for $g\rightarrow 0$, the gravitational coupling *g* has to be strong in order to allow for $\mathfrak {F}<0$. Hence, due to the generic flow of *g* depicted in Fig. 4 (right panel) this is the case only in the deep UV.

### The sign of $\mathfrak {F}$

The sign of $\mathfrak {F}$ in the deep UV is not fixed for general regulators. However, we will show that definite statements about the latter are indeed possible once a regulator is chosen. In the following, we consider $\sqrt{x}\,r^{\psi }_k(x)=(1-\sqrt{x})\theta (1-x)$ and $x\,r_k^h(x)=(1-x)\theta (1-x)$, which allow for analytic flow equations. For these choices, $\mathfrak {F}$ is given by

26 $$\begin{aligned} \mathfrak {F}=\frac{g}{70\pi } \left( \frac{448-79 \eta _h(k^2)}{8 (1 {+}\mu )^2}-\frac{7-2 \eta _\psi (k^2)}{1+\mu }\right) {+}\eta _\psi (0)+1, \end{aligned}$$

where we have moved the anomalous dimensions out of the loop integrals at momenta at which the integrand is peaked, i.e. $q^2=k^2$. This is shown to be a good approximation in [2, 8]. Now we investigate, for which values of $1+\mu$, $\mathfrak {F}$ does become zero, and, thus where $\mathfrak {F}<0$ is in principle possible. For the following analysis, we make use of the bounds of the anomalous dimensions $\eta _\psi (p^2)<1$ and $\eta _h(p^2)<2$, which are necessary such that the corresponding class of regulators, (10), remains well behaved, for details see [2]. We will take $\eta _\psi (p^2)$ as momentum independent and write $\eta _\phi =\eta _\phi (p^2)$. Furthermore, we assume that *g* does not take large values $g\lessapprox 10$ along the flow from $k=\infty$ to $k\rightarrow 0$. This assumption is supported by the FRG studies of pure quantum gravity and quantum gravity with the given matter content $N_f=1$ known to the authors. Summarising the main steps, we solve $\mathfrak {F}=0$ for $1+\mu$, where $\mathfrak {F}$ given in (26). Employing the approximation for $\eta _\psi (p^2)=\eta _\psi$ and the regulator constraints first leads to

27 $$\begin{aligned} -1<\eta _\psi <1. \end{aligned}$$

The assumption that *g* does not become large, implies that $\eta _\psi$ must be very close to the lower limit in (27). We express this finding as

28 $$\begin{aligned} 1+\eta _\psi =:\delta \ll 1, \end{aligned}$$

which allows one to expand the equation for $1+\mu$ in a Taylor series in $\delta$. More details can be found in Appendix A. The analysis results in a lower bound for $1+\mu$, namely

29 $$\begin{aligned} 1+\mu>\frac{145}{36}>4. \end{aligned}$$

Since all analyses of the UV behaviour of quantum gravity with non-zero $\eta _\psi$ report UV values of $1+\mu$ considerably smaller than 4, we conclude that the condition (29) is not fulfilled in the UV. In the IR, where (29) can hold, (28) is not fulfilled, since $\eta _\psi \ll 1$ and, thus, $\delta \approx 1$.

Truncations with zero fermion anomalous dimension $\eta _\psi =0$ [31, 38] require very large values of *g* of the order of $10^2$ in order to flip the sign of $\mathfrak {F}$, which we regard as unphysical.

In summary, we find that the flipping of signs for the critical exponents $\theta _i$ does not take place, as long as certain consistency conditions for the anomalous dimensions and the size of *g* are met. Thus, the flipping of signs of the critical exponents lies within a region in parameter space which is not reached by the flow. We conjecture that this is also true for more general classes of regulators. This, however, can presumably be verified only numerically.

## Multiple fermions

In this section we analyse whether the mechanism that prevents chiral symmetry breaking in the 4-fermion system is a property of the fermion number $N_f=1$ discussed above. In order to understand the impact of $N_f$ on the previous results, we consider a Fierz-complete model with $N_f$ fermions and $\text {SU}(N_f)\times \text {SU}(N_f)$ chiral symmetry. This model was also studied in a similar spirit in [38]. There chiral symmetry breaking was tested with a fermion anomalous dimension that was treated non-dynamically, as an input parameter. Here, we put forward a complete analysis of the fixed-point annihilation with dynamical anomalous dimensions and general regulators. The Fierz-complete fermion potential $V_{N_f}$ is given by

30 $$\begin{aligned} V_{N_f}(\psi ,\bar{\psi })= & {} -\bar{\lambda }_{-}\left[ ( \bar{\psi }^i\gamma _\mu \psi ^i)^2+(\bar{\psi }^i \gamma _\mu \gamma _5\psi ^i)^2\right] \nonumber \\&-\bar{\lambda }_{+}\left[ (\bar{\psi }^i\gamma _\mu \psi ^i)^2 +(\bar{\psi }^i\gamma _\mu \gamma _5\psi ^i)^2\right] . \end{aligned}$$

This model exhibits four fixed points. For $g=0$ one of them is in the fully IR attractive Gaussian fixed point. The second and third fixed points exhibit one IR attractive and one repulsive direction. The fourth fixed point is fully IR repulsive. Along the same lines as before we derive conditions for chiral symmetry breaking from a two-step procedure: We first analyse the possibility of fixed-point annihilation followed by a brief analysis of their stability.

### Fixed-point annihilation

The flow equations for the present $\text {SU}(N_f)\times \text {SU}(N_f)$ chirally symmetric NJL-type interacting fermion model, (31), are very similar to the previous ones. The purely fermionic part agrees with [54] and, when employing the Litim-type regulators given above, with [38]. However, due to a slightly different choice of the graviton-gauge ($\beta =1$ here, and $\beta =0$ in [38]), the contributions involving gravity interactions differ slightly. The $\beta$-functions are given by are given by

31 $$\begin{aligned} \beta _{\lambda _-}&= -2(N_f-1)\mathfrak {h}\lambda _-^2+2(1+\eta _\psi (0)+ \mathfrak {f})\,\lambda _-\nonumber \\ {}&\quad \, -2N_f\,\mathfrak {h}\lambda _+^2 +\frac{1}{4}\mathfrak {g}, \nonumber \\ \beta _{\lambda _+}&= -6\mathfrak {h}\lambda _+^2+2(1+\eta _\psi (0)\nonumber \\&\quad \,+\mathfrak {f}-2(N_f+1)\, \mathfrak {h}\lambda _-)\lambda _+ -\frac{1}{4}\mathfrak {g}. \end{aligned}$$

We observe that in the model with $N_f$ fermions, the fully IR attractive Gaussian fixed point potentially annihilates with one of the semi-stable fixed points, which was also true for the NJL-model discussed above. Additionally, the new fully IR repulsive (fully UV attractive) fixed point potentially annihilates with the other semi-stable fixed point. In both cases, the annihilation of the respective fixed points changes the topology of the phase diagram and possibly spoils the well-defined UV and IR limits. This potentially leads to chiral symmetry breaking during the flow. Remarkably, the fixed-point equation, Eq. (31) = 0, can, again, be solved in a simple closed form which is given in Appendix B. From the arguments of the corresponding square roots we derive two inequalities that are characteristic for the annihilation of the fixed points involved. They are given by

32 $$\begin{aligned}&\underbrace{\mathfrak {gh}(2 N_f-1)}_{>0}+\underbrace{2 \mathfrak {F}^2}_{\ge 0} \overset{!}{<} 0,\nonumber \\&\underbrace{\mathfrak {gh}\mathfrak {G} (N_f-1)}_{\ge 0}+2 \underbrace{\mathfrak {F}^2 (N_f+3)^2}_{\ge 0} \overset{!}{<} 0, \end{aligned}$$

where $\mathfrak {G} = (9 + 4N_f + 3N_f^2)>0$. With the same arguments as before it can be verified that the two inequalities (32), can both not be fulfilled individually for any choice of $N_f\ge 1$. Thus, also in this case the annihilation of fixed points is avoided on general grounds by the structure of the 4-fermion interaction.

### Stability of fixed points

The calculation of the fixed points’ stability for arbitrary $N_f$ is extensive and, furthermore, strongly dependent on the $N_f$-behaviour of the minimally coupled sub-system. A detailed, $N_f$-dependent stability analysis is therefore beyond the scope of this work. In the following, we consider the limiting cases $N_f\approx 1$ and $N_f\rightarrow \infty$. The former case is identical to the analysis of the NJL-model with one fermion discussed above, where we noted the significance of the sign of $\mathfrak {F}$. Hence, we now study the stability of the fixed point of the system with $N_f$ fermions in a large-$N_f$-expansion. In [2] the minimally coupled gravity-fermion system has been studied. Let us first consider the approximation where the contributions of the anomalous dimensions are neglected. Then the minimally coupled sub-system reveals an asymptotic scaling of the UV fixed-point value of *g*, $g^*$, as $g^*\sim 1/N_f$ for $N_f\rightarrow \infty$, [2]. This motivates a rescaling of the couplings according to

33 $$\begin{aligned} (g,\lambda _i) \rightarrow (\tilde{g}= N_f g, \tilde{\lambda }_i = N_f \lambda _i), \end{aligned}$$

such that $\tilde{g}^*$ approaches a finite value as $N_f\rightarrow \infty$. Expanding the fixed points for the resulting system in $1/N_f$ we find

34 $$\begin{aligned} \mathcal {\tilde{F}}_{1/2}&\rightarrow \left\{ \mp \frac{\mathfrak {\tilde{g}}}{8 N_f},\,\frac{1}{2\mathfrak {h}} \left( 1+\frac{1+\mathfrak {f}}{N_f} \mp \left( 1+\frac{4(1+\mathfrak {f})+\mathfrak {h\tilde{g}}}{4N_f}\right) \right) \right\} ,\nonumber \\ \mathcal {\tilde{F}}_{3/4}&\rightarrow \left\{ \mp \frac{1}{2\mathfrak {h}}\left( 1+\frac{2+4 \mathfrak {f}+ \mathfrak {h \tilde{g}}}{4 N_f}\right) ,\, \frac{1}{2\mathfrak {h}}\left( 1-\frac{1+\mathfrak {f}}{N_f}\pm \frac{6+\mathfrak {h \tilde{g}}}{4 N_f}\right) \right\} , \end{aligned}$$

where $\mathfrak {\tilde{g}}=\mathfrak {g}(\tilde{g})=N_f^2\,\mathfrak {g}(g)$; see Eq. (21). For $N_f\rightarrow \infty$ the gravity contributions in (34) tend towards zero. Then only diagrams with purely fermionic loops contribute to the flow. All gravity contributions are sub-leading and the flow of the 4-fermion couplings is independent of the rest of the flow. In this limit we arrive at the fixed points $\mathcal {\tilde{F}}_{1/2}\sim \{0,\frac{1}{2\mathfrak {h}}(1\mp 1)\}$ and $\mathcal {\tilde{F}}_{3/4}\sim \{\mp \frac{1}{2\mathfrak {h}},\frac{1}{2\mathfrak {h}}\}$. Note that $\mathcal {\tilde{F}}_1$ is the Gaussian fixed point here. The critical exponents also reflect this gravitational decoupling, and are given in Appendix C to the order $\mathcal {O}(1/N_f)$. Hence, the scaling $g^*\sim 1/N_f$ leads to the expected behaviour in the large $N_f$-limit: the large number of fermions dominates the fluctuation physics.

However, the situation is more complicated in the full system with momentum-dependent anomalous dimensions. Then the minimally coupled sub-system exhibits an asymptotic scaling of approximately $g^*\sim 1/\sqrt{N_f}$, [2], in contradistinction to the $1/N_f$ discussed above. In this case, the large-$N_f$-limit is non-trivial and fingerprints of the gravitational interactions survive the fermionic dominance. This is readily verified by replacing $\mathfrak {\tilde{g}} \rightarrow N_f \mathfrak {\tilde{g}}$ in (34) in order to account for the modified asymptotic $N_f$-scaling. This leads to

35 $$\begin{aligned} \mathcal {\tilde{F}}_{1/2}&\rightarrow \left\{ \mp \frac{\mathfrak {\tilde{g}}}{8},\,\frac{1}{2\mathfrak {h}}\left( 1\mp \left( 1+\frac{\mathfrak {h \tilde{g}}}{4}\right) \right) \right\} ,\nonumber \\ \mathcal {\tilde{F}}_{3/4}&\rightarrow \left\{ \mp \frac{1}{2\mathfrak {h}}\left( 1+\frac{\mathfrak {h \tilde{g}}}{4}\right) ,\,\frac{1}{2\mathfrak {h}}\left( 1\pm \frac{\mathfrak {h \tilde{g}}}{4}\right) \right\} , \end{aligned}$$

in the limit $N_f\rightarrow \infty$. A more detailed analysis of the $g^*\sim 1/\sqrt{N_f}$-scaling reveals that these remainders of gravity do, however, not allow for chiral symmetry breaking in the large-$N_f$-limit.

This discussion shows that the scaling of $g^*$ for a large number of matter fields is of crucial importance for the properties of the combined theory. In particular, it was found in [2] that the fixed-point value $g^*$ grows as the number of scalar fields, $N_s$, increases, which is in sharp contrast to the fermionic case discussed here. It is therefore tempting to study the flow of scalar self-interactions (see also [39]), at moderate $N_s$ taking into account the corresponding scaling of $g^*$.

The current analysis suggests that gravity plays no or only a negligible rôle in the large-$N_f$-limit and chiral symmetry breaking is absent. Coming back to our original problem, we conclude that the sign of $\mathfrak {F}$ is of interest only at low and intermediate $N_f$, where the gravitational contributions are not suppressed by fermion fluctuations. Only in this regime can negative $\mathfrak {F}$ spoil the stability of the fixed points. Such a negative $\mathfrak {F}$ requires $(1+\mu )>4$, as discussed above. However, in [2] it has been shown that $1+\mu$ stays well below this limit for all $N_f$ and approaches 0 as $N_f\rightarrow \infty$. We conclude that chiral symmetry breaking is absent not only in the present combination of a general interaction 4-fermion model and the asymptotically safe minimally coupled gravity-fermion sub-system put forward in [2]. Instead this analysis implies that all combinations of the present 4-fermion model together with arbitrary minimally coupled gravity-fermion sub-systems avoid chiral symmetry breaking, provided these sub-systems exhibit the following properties: (i) They are asymptotically safe. (ii) They ensure $1+\mu <4$ along the whole RG-trajectory. (iii) They allow for a large-$N_f$-limit where $g^*\rightarrow 0$ as $N_f\rightarrow \infty$ with a scaling $g^*\sim 1/\sqrt{N_f}$ or faster.

## Conclusions

We have analysed the possibility of chiral symmetry breaking in theories of interacting fermions and quantum gravity using a self-consistent FRG-approach. As a general feature for models with 4-fermion interaction of the NJL-type we found that chiral symmetry breaking is absent irrespective of the number of fermion fields and mostly independent of the regularisation scheme.

For this analysis, we have reduced the condition for the existence of well-defined UV and IR limits to the existence and stability of fixed points of the renormalisation group flow. We have interpreted the possible annihilation as well as the change of stability of the latter as indications for a topological change in the phase diagram of the 4-fermion couplings, which could lead to chiral symmetry breaking in the UV. We have found that fixed-point annihilation is ruled out generally for the models discussed here independent of the chosen regulator and the fermion number. However, the change of attractivity of the fixed points is more subtle in particular for an arbitrary number of fermions. Still, we were able to argue that in neither of the present models we expect the fixed-point-attractivity to change due to the interacting of interacting fermions with gravity. This conclusion is drawn from a more detailed analysis based on a specific choice of regulator in the limits $N_f\approx 1$ and $N_f\rightarrow \infty$. In the latter case, the flow of the 4-fermion couplings decouples either completely or at least mostly from the rest of the fermion–gravity system. The degree of the latter decoupling depends on the scaling of the fixed-point value $g^*$ as $N_f\rightarrow \infty$. Our results imply that gravity-induced chiral symmetry breaking at the Planck scale is avoided for a general class of models with chirally symmetric 4-fermion interactions.

## Sign of $\mathfrak {F}$ as a function of $\mu$

In the following, we analyse in detail the sign of $\mathfrak {F}=1+\eta _\psi (0)+\mathfrak {f}$ for a Litim-type regulator. The explicit expression is given in (26). For vanishing gravitational constant $g\rightarrow 0$ we have $\mathfrak {F}\rightarrow 1$. Hence for small values of *g* we will have $\mathfrak {F}\approx 1$. We see that some of the critical exponents given in (25) change sign as soon as $\mathfrak {F}$ becomes negative. We now solve $\mathfrak {F}=0$ for $1+\mu$ for momentum-independent fermion anomalous dimensions $\eta _\psi (p^2)= \eta _\psi$. For the given class of regulators, (10), the $\eta$’s have to satisfy the bounds $\eta _\psi <1$ and $\eta _h<2$. Solving Eq. (26) = 0 we get

A1 $$\begin{aligned}&(1+\mu )_{1,2} = \frac{1}{140 \pi (1+\eta _\psi )}\left( \phantom {\sqrt{g \left( 35 \pi (1+\eta _\psi ) (79 \eta _h-448)+(7-2 \eta _\psi )^2 g\right) }}(7-2 \eta _\psi ) g \nonumber \right. \\&\quad \left. \pm \sqrt{g \left( 35 \pi (1+\eta _\psi ) (79 \eta _h-448)+(7-2 \eta _\psi )^2 g\right) }\right) . \end{aligned}$$

This equation only has real solutions if the argument in the square root is positive-semidefinite. Hence, in order to approach the limit $\mathfrak {F}\rightarrow 0$ for a real $\mu$ we need

A2 $$\begin{aligned} (7-2 \eta _\psi )^2 g\ge 35 \pi (1+\eta _\psi ) (448-79 \eta _h). \end{aligned}$$

The left-hand side is positive-semidefinite. Hence, we see that for $\eta _\psi \le -1$ the above inequality is satisfied for $\eta _h<2$. However, $1+\eta _\psi \rightarrow 0$ leads to a solution of (A1) where $1+\mu \rightarrow \infty$. This means that the flow would need to pass the graviton-propagator pole in order to reach the regime $\eta _\psi <-1$. This transition, however, is not admitted by the flow equations. Therefore, we conclude $-1<\eta _\psi <1$. Then, for $\eta _h<2$, we find that the right-hand side of (A2) is positive, and we write

A3 $$\begin{aligned} g>35 \pi (448-79 \eta _h) \frac{1+\eta _\psi }{ (7-2 \eta _\psi )^2}>10150 \pi \frac{1+\eta _\psi }{ (7-2 \eta _\psi )^2}. \end{aligned}$$

Due to $-1<\eta _\psi <1$ we have

A4 $$\begin{aligned} 0<\frac{1+\eta _\psi }{ (7-2 \eta _\psi )^2}<\frac{2}{25}. \end{aligned}$$

Typical values of *g* in the UV are of order 1, and we restrict ourselves to couplings $g\lessapprox 10$. The numerator $(1+\eta _\psi )$ in (A3) has to become very small and, in particular, considerably smaller than *g*. This is expressed as

A5 $$\begin{aligned} (1+\eta _\psi )=:\delta \ll 1. \end{aligned}$$

For small $\delta$ we can expand (A1) in $\delta$ and get, keeping only the lowest orders in $\delta$,

A6 $$\begin{aligned} (1+\mu )_1&\approx \frac{9 g}{70 \pi \delta }, \end{aligned}$$

A7 $$\begin{aligned} (1+\mu )_2&\approx \frac{56}{9}-\frac{79 \eta _h}{72}>\frac{145}{36}>4. \end{aligned}$$

We have used $\eta _h<2$ to arrive at the lower bound for $(1+\mu )_2$. Now, using Eq. (A3) and expanding in $\delta$ we get a lower bound for $(1+\mu )_{1}$, which reads

A8 $$\begin{aligned} (1+\mu )_{1}>140, \end{aligned}$$

to lowest order in $\delta$. Thus, respecting the regulator bound, we must have

A9 $$\begin{aligned} (1+\mu )>4, \end{aligned}$$

in order for $\mathfrak {F}$ to become negative.

## Fixed points for $N_f$ fermions

The fixed-point equation, Eq. (31) = 0, can be solved in a simple and closed form for $(\lambda ^*_-,\lambda ^*_+)$ for an arbitrary number of fermions. The solutions are given by the four fixed points $\mathcal {F}^{(N)}_{1\le i\le 4}$ to wit

B1 $$\begin{aligned} \mathcal {F}^{(N)}_{1/2}=&\left( -\frac{2 \mathfrak {F}\mp \sqrt{4 \mathfrak {F}^2+2 (2N_f-1) \mathfrak {h}\mathfrak {g}}}{4 (2N_f-1) \mathfrak {h}},\frac{\mp 2 \mathfrak {F}+\sqrt{4 \mathfrak {F}^2+2 (2N_f-1) \mathfrak {h}\mathfrak {g}}}{4 (2N_f-1) \mathfrak {h}}\right) ,\nonumber \\ \mathcal {F}^{(N)}_{3/4}=&\left( \frac{2 \mathfrak {F}(N_f+3)\pm \sqrt{4 (N_f+3)^2 \mathfrak {F}^2+ 2 (N_f-1) \mathfrak {h}\mathfrak {g} \mathfrak {G}}}{4 \mathfrak {h}\mathfrak {G}},\right. \nonumber \\&\left. \right. \left. \frac{2 \mathfrak {F}(\mathfrak {G}{+}(N_f{-}1) N_f)\mp (N_f{+}3) \sqrt{4 (N_f{+}3)^2 \mathfrak {F}^2{+} 2 (N_f{-}1) \mathfrak {h}\mathfrak {g} \mathfrak {G}}}{4 \mathfrak {h}\mathfrak {G}(N_f{-}1)}\right) . \end{aligned}$$

Without gravity (where $g=0$), we have $(\mathfrak {f},\mathfrak {g})\rightarrow (0,0)$ and hence $\mathcal {F}_1^{(N)}\rightarrow (0,0)$ is the Gaussian fixed point. For $g\ne 0$ all fixed points are non-Gaussian.

## Critical exponents in the large-$N_f$-limit

The critical exponents for the rescaled couplings $(\tilde{\lambda }_\sigma ,\tilde{\lambda }_v)$ for large $N_f$ take the form

C1 $$\begin{aligned} \tilde{\theta }_{1/3}&\approx \left\{ \pm (2+\frac{2 \mathfrak {\tilde{f}}}{N_f}),2+\frac{2+2 \mathfrak {\tilde{f}}+ \mathfrak {h} \mathfrak {\tilde{g}}}{N_f}\mp \frac{2}{N_f}\right\} ,\nonumber \\&\tilde{\theta }_{4} \approx \left\{ -2 -\frac{2 \mathfrak {\tilde{f}}+\mathfrak {h} \mathfrak {\tilde{g}}}{N_f},2 +\frac{2 \mathfrak {\tilde{f}}+4}{N_f}\right\} , \nonumber \\ \tilde{\theta }_{2}&\approx \left\{ -2-\frac{8+4 \mathfrak {\tilde{f}}+\mathfrak {h} \mathfrak {\tilde{g}}+\sqrt{64-\mathfrak {h}^2 \mathfrak {\tilde{g}}^2}}{2 N_f},\right. \nonumber \\&\left. -2-\frac{8+4 \mathfrak {\tilde{f}}+\mathfrak {h} \mathfrak {\tilde{g}}-\sqrt{64-\mathfrak {h}^2 \mathfrak {\tilde{g}}^2}}{2 N_f}\right\} , \end{aligned}$$

where $\mathfrak {\tilde{f}}=\mathfrak {f}(\tilde{g})$. To leading order in $1/N_f$, the gravity contributions decouple from the 4-fermion model.

## References

[1] Christiansen N, Knorr B, Meibohm J, Pawlowski JM, Reichert M (2015). Phys. Rev. D.

[2] Meibohm J, Pawlowski JM, Reichert M (2016). Phys Rev D.

[3] S. Weinberg, in *General Relativity: An Einstein Centenary Survey*, ed. by S.W. Hawking, W. Israel (Cambridge University Press, Cambridge, 1979), p. 790

[4] Reuter M (1998). Phys. Rev. D.

[5] Souma W (1999). Prog. Theor. Phys..

[6] Reuter M, Saueressig F (2002). Phys. Rev. D.

[7] Christiansen N, Litim DF, Pawlowski JM, Rodigast A (2014). Phys. Lett. B.

[8] Christiansen N, Knorr B, Pawlowski JM, Rodigast A (2016). Phys. Rev. D.

[9] Lauscher O, Reuter M (2002). Phys. Rev. D.

[10] Codello A, Percacci R (2006). Phys. Rev. Lett..

[11] Codello A, Percacci R, Rahmede C (2008). Int. J. Mod. Phys. A.

[12] Codello A, Percacci R, Rahmede C (2009). Ann. Phys..

[13] Machado PF, Saueressig F (2008). Phys. Rev. D.

[14] Benedetti D, Machado PF, Saueressig F (2009). Mod. Phys. Lett. A.

[15] Eichhorn A, Gies H, Scherer MM (2009). Phys. Rev. D.

[16] Manrique E, Rechenberger S, Saueressig F (2011). Phys. Rev. Lett..

[17] Rechenberger S, Saueressig F (2012). Phys. Rev. D.

[18] I. Donkin, J.M. Pawlowski (2012). arXiv:1203.4207 [hep-th]

[19] Codello A, D’Odorico G, Pagani C (2014). Phys. Rev. D.

[20] K. Falls, D. Litim, K. Nikolakopoulos, C. Rahmede (2013). arXiv:1301.4191 [hep-th]

[21] Falls K (2016). JHEP.

[22] K. Falls, D.F. Litim, K. Nikolakopoulos, C. Rahmede (2014). arXiv:1410.4815 [hep-th]

[23] Falls K (2015). Phys. Rev. D.

[24] Gies H, Knorr B, Lippoldt S (2015). Phys. Rev. D.

[25] H. Gies, B. Knorr, S. Lippoldt, F. Saueressig (2016). arXiv:1601.01800 [hep-th] 10.1103/PhysRevLett.116.211302 27284643

[26] Niedermaier M, Reuter M (2006). Living Rev. Relativ..

[27] R. Percacci, in *Approaches to Quantum Gravity* 111–128*, ed. by D. Oriti (2007). arXiv:0709.3851 [hep-th]

[28] Litim DF (2011). Philos. Trans. R. Soc. Lond..

[29] Reuter M, Saueressig F (2012). New J. Phys..

[30] Dou D, Percacci R (1998). Class. Quant. Grav..

[31] Percacci R, Perini D (2003). Phys. Rev. D.

[32] Percacci R, Perini D (2003). Phys. Rev. D.

[33] Folkerts S, Litim DF, Pawlowski JM (2012). Phys. Lett. B.

[34] Donà P, Percacci R (2013). Phys. Rev. D.

[35] Donà P, Eichhorn A, Percacci R (2014). Phys. Rev. D.

[36] Donà P, Eichhorn A, Percacci R (2015). Can. J. Phys..

[37] Donà P, Eichhorn A, Labus P, Percacci R (2016). Phys. Rev. D.

[38] Eichhorn A, Gies H (2011). New J. Phys..

[39] Eichhorn A (2012). Phys. Rev. D.

[40] Henz T, Pawlowski JM, Rodigast A, Wetterich C (2013). Phys. Lett. B.

[41] K.-Y. Oda, M. Yamada (2015). arXiv:1510.03734 [hep-th]

[42] A. Eichhorn, A. Held, J.M. Pawlowski (2016). arXiv:1604.02041 [hep-th]

[43] Wetterich C (1993). Phys. Lett. B.

[44] Litim DF, Pawlowski JM (1998). Phys. Lett. B.

[45] Weldon HA (2001). Phys. Rev. D.

[46] Gies H, Lippoldt S (2014). Phys. Rev. D.

[47] Lippoldt S (2015). Phys. Rev. D.

[48] Mitter M, Pawlowski JM, Strodthoff N (2015). Phys. Rev. D.

[49] H. Gies, J. Jaeckel, Eur. Phys. J. C **46**, 433 (2006). arXiv:hep-ph/0507171 [hep-ph]

[50] Scherer MM, Gies H, Rechenberger S (2009). Acta Phys. Polon. Supp..

[51] Braun J (2012). J. Phys..

[52] Gies H, Rechenberger S, Scherer MM, Zambelli L (2013). Eur. Phys. J. C.

[53] Litim DF (2000). Phys. Lett. B.

[54] Gies H, Jaeckel J, Wetterich C (2004). Phys. Rev. D.

